# Mixture effects of co-formulants and two plant protection products in a liver cell line

**DOI:** 10.17179/excli2022-5648

**Published:** 2023-02-13

**Authors:** Katreece Feiertag, Mawien Karaca, Benjamin Fischer, Tanja Heise, Denise Bloch, Tobias Opialla, Tewes Tralau, Carsten Kneuer, Philip Marx-Stoelting

**Affiliations:** 1 German Federal Institute for Risk Assessment, Department Pesticides Safety, Berlin, Germany

**Keywords:** mixture effects, plant protection product, liver toxicity, co-formulants, Cytochrome P450 enzymes

## Abstract

Plant protection products (PPPs) consist of one or more active substances and several co-formulants. Active substances provide the functionality of the PPP and are consequently evaluated according to standard test methods set by legal data requirements before approval, whereas co-formulants' toxicity is not as comprehensively assessed. However, in some cases mixture effects of active substances and co-formulants might result in increased or different forms of toxicity. In a proof-of-concept study we hence built on previously published results of Zahn et al. (2018[38]) on the mixture toxicity of Priori Xtra^®^ and Adexar^®^ to specifically investigate the influence of co-formulants on the toxicity of these commonly used fungicides. Products, their respective active substances in combination as well as some co-formulants were applied to human hepatoma cell line (HepaRG) in several dilutions. Cell viability analysis, mRNA expression, abundance of xenobiotic metabolizing enzymes and intracellular concentrations of active substances determined by LC-MS/MS analyses demonstrated that the toxicity of the PPPs is influenced by the presence of co-formulants *in vitro*. PPPs were more cytotoxic than the mix of their active substances. Gene expression profiles of cells treated with the PPPs were similar to those treated with their respective mixture combinations with marked differences. Co-formulants can cause gene expression changes on their own. LC-MS/MS analyses revealed higher intracellular concentrations of active substances in cells treated with PPPs compared to those treated with the respective active substances' mix. Proteomic data showed co-formulants can induce ABC transporters and CYP enzymes. Co-formulants can contribute to the observed increased toxicity of PPPs compared to their active substances in combination due to kinetic interactions, necessitating a more comprehensive evaluation approach.

## Abbreviations

ACTB human actin, beta

B2M human beta-2-microglobulin

CE collision energy

CT cycle threshold

CUR curtain gas

CXP collision cell exit potential

DMEM Dulbecco's Modified Eagle Medium

DMSO dimethyl sulfoxide

DP declustering potential

DPH 1,6-diphenyl-1,3,5-hexatriene

EC_50_ median effective concentration

EP entrance potential

FA formic acid

FCS fetal calf serum

GAPDH human glyceraldehyde-3-phosphate dehydrogenase

GS1 nebulizing gas/ion source gas 1

GS2 drying gas/ion source gas 2

HKG housekeeping gene

HPRT1 human hypoxanthine phosphoribosyl transferase 1

IS ion spray voltage

MDR1 multidrug resistance protein 1

MRP2 multidrug resistance-associated proteins 2

MSDS material safety data sheet

NOAEL no observed adverse effect level

NRU neutral red uptake

PBS phosphate-Buffered Saline

Pgp P-glycoproteins

POE polyoxyethylene

PPP plant protection product

QuEChERS Quick, Easy, Cheap, Effective, Rugged and Safe

REACH Registration, Evaluation, Authorisation and Restriction of Chemicals

WST-1 water soluble tetrazolium

RLP0 human ribosomal protein, large

p0 r-value, anisotropy value

SD standard deviation

TEM temperature of ion source

## Introduction

PPPs are mixtures used in agriculture to protect desirable plants from pest organisms and to decrease or prevent the growth of undesirable plants (European Commission 1995-2022[12]; Kraehmer et al., 2014[22]; Popp et al., 2013[29]) resulting in increased crop yields worldwide (Oerke and Dehne, 2004[26]; Tudi et al., 2021[34]). 

PPPs are generally mixtures containing one or more active substances as well as several co-formulants. Active substances provide the functionality of the PPPs, whereas co-formulants improve its applicability (i.e. solvents, anti-foaming-agents, wetting agents) (Hazen, 2000[15]). Active substances are therefore assumed to contribute to PPP's total toxicity and are thus evaluated according to standard test methods set by legal data requirements before approval. The authorization of active substances as well as PPPs is set in Regulation (EC) No 1107/2009 (European Commission, 2009[10]). Individual active substances are evaluated extensively prior to approval e.g. for acute-, short- and long-term toxicity, genotoxicity developmental and reproductive toxicity (EU No 283/2013 European Commission, 2013[11]). However, only acute toxicity testing is required for the authorization of the PPPs with limited endpoints like mortality, clinical symptoms and pathology. Repeated dose toxicity studies are not required, thus possible sub-chronic effects and endpoints generally not examined in acute studies (i.e. hematology, clinical chemistry and histopathology) are not investigated (Commission Regulation (EC) No 1107/ 2009[10]; European Commission, 2021[12]). On the other hand, the relevance of co-formulants for toxicity is not that well understood. Co-formulants produced in larger volumes may be regulated by REACH (the EU chemicals regulation) and toxicological data may be available for individual co-formulants. But in general, co-formulants in the EU neither require toxicological evaluation nor authorization under EU regulation 1107/ 2009. However, a list of co-formulants not acceptable for inclusion in PPPs is available as an amendment to Regulation (EC) No 1107/2009 ANNEX III. This list has been recently updated to include polyethoxylated tallowamines (Commission regulation (EU) 2021/383; European Commission, 2021[12])) as several studies have shown that the co-formulant tallowamine used in glyphosate containing products exhibits toxic effects (Chaufan et al., 2014[5]; Coalova et al., 2014[7]; Kim et al., 2013[21]; Song et al., 2012[32]). Several other studies have shown that, co-formulants can exhibit toxic effects of their own (Li et al., 2015[24]; Song et al., 2012[33]). 

In addition, mixture effects have been reported between active substances. A recent study on active substances' mixtures showed that a hepatotoxic compound's effect was potentiated by a non-hepatoxic compound on HepaRG cells *in vitro* (Lasch et al., 2021[23]). Mixture effects of active substances have also been showed *in vivo* (Pascotto et al., 2015[28]; Rieke et al., 2017[31]). Other studies have investigated the mixture effects caused by the interaction of co-formulants and active substances, showing that co-formulants can indeed cause differential toxicity when in combination (Li et al., 2015[24]). Co-formulants' differential toxicity has been linked to toxicokinetic interactions between co-formulants and active substances *in vitro* (Karaca et al., 2021[20]). 

PPPs may exhibit increased toxic effects compared to the individual active substances used therein as shown by several *in vitro* studies (Holeckova et al., 2013[17]; Mesnage et al., 2014[25]; Zahn et al., 2018[38]). 

In a previous study we investigated two PPPs, Priori Xtra^®^ and Adexar^®^, both containing two active substances, we compared the product to the individual active substances as well as to the combination of the active substances (Zahn et al., 2018[38]). The composition including the declared co-formulants according to the Material Safety Data Sheet is given in Table 1(Tab. 1). All four active substances have known effects on the liver, leading to hypertrophy (Germany, 2013[14]; EFSA, 2015[8]; Heise et al., 2015[16]; Ireland, 2010[18]; UK, 2009[36], 2011[35]). This adverse outcome is related to the activation of nuclear receptors, like PXR (Pregnane-X-Receptor), CAR (Constitutive Androstane Receptor) and AhR (Aryl hydrocarbone Receptor) and the increased synthesis of xenobiotic metabolizing enzymes (Elcombe et al., 2010[9]; Wei et al., 2000[37]; Zhang et al., 2015[39]). In the work by Zahn et al. we found increased cytotoxicity of the products as compared to the active substances in human liver derived HepG2 und HepaRG cell lines as well as alterations in gene expression profiles especially in genes coding for receptor-dependent xenobiotic metabolizing enzymes. In the present study, we follow-up on the role individual co-formulants may play, explaining the effects described in the previous study. To this end, we focused on identifying toxic co-formulants and characterizing their kinetic impact. 

### Test substances

The test substances cyproconazole [CAS # 94361-06-5; Syngenta; Batch # CHF1E00042; purity 96.8 %] and epoxiconazole [Cas # 133855-98-8; BASF; Batch # 8563; purity 97.0 %] were obtained directly from the producing companies in technical quality (the same quality the substances are used in PPPs). The test substances fluxapyroxad [CAS # 907204-31-3; Batch # SZBF160XV; purity 99.9 %] and azoxystrobin [CAS # 131860-33-8; Batch # BCBT1118V; purity ≥ 98 %] were obtained from Sigma-Aldrich (Taufkirchen, Germany). The PPPs Adexar^®^ (registration no. 006958-00; BASF; Batch # 0013044538; 62.5 g/l epoxiconazole; 62.5 g/l fluxapyroxad) and Priori Xtra® (registration no. 005481-00; Syngenta; Batch # 139731; 200 g/l azoxystrobin; 80 g/l cyproconazole) were obtained commercially in Germany. The co-formulants 2-hydroxy-N,N-dimethylpropanamide (Lot# CDS021721), dipropylene glycol (Lot# BCBS8922V, CAS: 25265-71-8), propylene glycol (Lot# LRAB0286, CAS: 57-55-6), Eumulgin® B25 (Lot# BCBV7832, CAS: 68439-49-6), nanoclay, hydrophilic bentonite (Lot# MKCF4495, CAS: 1302-78-9), xanthan from xanthomonas campestris (Lot# BCBV5654, CAS: 1302-78-9), polymethacrylate, N,N-dimethyldecanamide (Lot# CDS001534) were obtained from Sigma-Aldrich (Taufkirchen, Germany). 2-ethylhexyl S lactate (Batch# 1702000034) was obtained from CorbionPurac (Amsterdam, Netherlands). 

All substances or products were diluted in DMSO, resulting in a final DMSO medium concentration of 0.4 % in all exposed wells. All other chemicals were purchased from Merck (Darmstadt, Germany) or Sigma-Aldrich (Taufkirchen, Germany) in the highest available purity.

### Cell culture

The HepaRG cells were obtained from Biopredic International (Saint Grégoire, France) and were grown for two weeks in Williams medium (Pan-Biotech GmbH, Aidenbach, Germany) containing 10 % fetal calf serum (FCS) (PAN-Biotech GmbH, Aidenbach, Germany), 100 U/ml penicillin, 100 µg/ml streptomycin, 0.05 % human insulin (PAA Laboratories GmbH, Pasching, Austria) and 50 µM hydrocortisone-hemisuccinate (Sigma-Aldrich, Taufkirchen, Germany). For the differentiation, cells were incubated for a further two weeks in differentiation medium. In addition to the already mentioned components, the differentiation medium contained 1.7 % DMSO. HepaRG cells were treated with phenol-red-free Williams medium (Pan-Biotech GmbH, Aidenbach, Germany) which contains all ingredients as the proliferation medium, but only 2 % FCS. Cells were incubated at 37° C in a 5 % CO_2 _/ 5 % humidity atmosphere in a Binder cell culture incubator. 

### Cytotoxicity measurement

NRU (neutral red assay) was used for cytotoxicity analysis of both PPPs and for the combination of the active substances according to the protocol by Repetto et al. (2008[30]). Before the neutral red assay was performed, HepaRG cells were seeded in 96-well plates (9x10^3 ^cells per well). The cells were cultured for 4 weeks including 2 weeks of proliferation followed by 2 weeks of differentiation. Cells were then treated with the PPPs, the active substances' mix, and several individual co-formulants for each PPP (for each, eight different concentrations in culture medium with a final solvent concentration of 0.4 % DMSO) for 24 h. The detergent Triton X-100 (0.01 %) served as positive control. After 24 h, treatment medium was removed and cells were washed once with 100 *μ*l PBS per well. Then, 100 μl neutral red medium was added per well and cells were incubated for 2 h at 37 °C. Afterwards, neutral red medium was removed and 150 μl of an acidic ethanol solution was added per well for extraction to remove the basic dye. Uptake of neutral red by endocytosis can be quantified by fluorescence measurement (Excision 530 nm, Emission 645 nm) using an Infinite M200pro plate reader (Tecan, Maennedorf, Switzerland). 

### Gene expression analysis

HepaRG cells were seeded in 6 well plates and allowed to proliferate and differentiate for 4 weeks. Total RNA was isolated from cells treated for 24 h with the two PPPs, their active substance mix and some of their co-formulants whilst ensuring a final concentration of 0.4 % DMSO in each well. The isolation was carried out using peqGOLD TriFast™ (peqlab, Erlangen, Germany) reagent according to the manufacturer's instructions. Quantity and quality of the RNA samples were measured using a Nanodrop spectrophotometer (NanoDrop 2000; Thermo Fisher Scientific, Waltham, MA, USA). For a ratio absorbance at 260/280 and 260/230 nm, values were accepted within the range of 1.8-2.2 and 1.6-2.2 nm respectively. As recommended by the supplier, all samples were purified prior to the RT2 Profiler PCR Array PAHS-3401Z procedures following the supplier's manual using the RNeasy® MiniElute Cleanup kit. RNA samples were then transcribed to cDNA according to the supplier's manual using the RT2 First Strand kit. Gene expression analysis was subsequently performed with RT2 Profiler PCR Array PAHS-3401Z kit in combination with RT² SYBR® Green qPCR mastermix (Qiagen®; Hilden, Germany) on an ABI 7900HT instrument (Applied Biosystems, Darmstadt, Germany). The cycling condition for PCR reaction was programmed according to the table provided by the supplier's manual. A table of CT values was created in an excel sheet and then uploaded to the data analysis web portal (http://www.qiagen.com/geneglobe). CT values were normalized based on a manual selection procedure. The data analysis web selected and displayed the standard HKG genes by default. Afterwards, normalization was done if the selected genes had only a small change in their expression across different sample groups. The selected HKG were ACTB, B2M, GAPDH, HPRT1, and RLP0. The data analysis web portal tool calculated fold change/regulation using the CT method, where delta CT was calculated between gene of interest (GOI) and an average of reference genes (HKG), followed by delta-delta CT calculations (delta CT (test group)-delta CT (control group)). Fold Change was then calculated using 2^ (-delta delta CT) formula. The data was downloaded as Excel sheets and PDF files. To decrease false-positives due to random variability, p-values were adjusted by FDR-method (Benjamini and Hochberg, 1995[4]). In addition, gene ontology (GO) term enrichment analysis based on R software was applied to identify the pathways in which differentially expressed genes (DEG) significantly enriched.

### Quantitative mass spectrometry-based immunoassays for CYP enzymes and transporters

HepaRG cells were cultured, allowing for their proliferation and differentiation for 4 consecutive weeks in 6-well plates (2x10^5^ cells per well). Cells were subsequently treated (with the formulations, their active substances in combination and some of their co-formulants) and incubated for 24 h at 37 °C. Cells were then lysed following the protocol provided by Signatope. The quantification of the Cyp enzymes and ABC transporters was carried out by Signatope with the use of quantitative mass spectrometry-based immunoassays. There, the amount of protein was determined, and the tryptic digestion of the lysates was carried out, followed by the addition of the isotope labeled signature peptides from the sequences of CYP450 enzymes and transporters. In a further step, these synthetic peptides and the endogenous peptides (originating from the sample) were enriched and precipitated with the aid of peptide group-specific antibodies. These peptides were eluted from the antibodies after precipitation and detected by mass spectrometry (LC-MS-MS QExactive, Thermo). The proteins were quantified indirectly by referencing the signal from the endogenous peptide to the signal from the synthetic peptide. 

### Sample preparation 

The extraction of the products and their active substances from HepaRG cells, PBS and the medium was done using the Quick, Easy, Cheap, Effective, Rugged and Safe (QuEChERS) technique with a few deviations from the original proposal by Anastassiades et al. (2003[1]). Sample preparation was as follows: Cell samples were dispersed in 10 ml of acetonitrile and Milli-Q water mix (4:1 ratio respectively) and placed in an ultrasonic bath for 15 min. Medium and PBS samples were directly sonicated for 15 min. For each sample 1 ml of the mixture was added to a 50 ml centrifuge tubes to which 4 ml of Milli-Q water was added; 10 ml of precooled acetonitrile (ACN) was added, and samples were vigorously shaken by a vortex mixer for 10 min. One Supel QuE Citrate Extraction Tube 55227-U (Sigma-Aldrich, Taufkirchen, Germany) was added to the samples and the centrifuge tubes were shaken immediately for one minute by hand. Centrifuge tubes were vigorously shaken by a vortex mixer for another 10 min. Centrifuge tubes were subject to centrifugation for 5 min at 3000 g and 4 °C. 7 ml of the supernatant was transferred to 15 ml centrifuge tubes from which the ACN was evaporated with a nitrogen stream EVA 1 Vis (VLM GmbH, Bielefeld, Germany). Residues were redissolved in 0.20 ml of the mobile phase (start conditions), mixed for 5 min and sonicated for 5 min, and then transferred to a vial for chromatographic analysis. 

### LC-MS/MS analysis 

Active substances were chromatographically separated on an Agilent 1260 series liquid chromatography system (Agilent Technologies, Heilbronn, Germany) consisting of a binary pump system (G1312B), degasser (G4225A), column oven (G1316A TCC), auto sampler with thermostat (G1367E HiP ALS + G1330B) and an Instant Pilot controller (G4208A). The separation was performed at 30 °C with a reversed-phase Luna^®^ C_8 _LC Column (150 x 2 mm, 5 particle size) Phenomenex, Aschaffenburg, Germany). The mobile phase had a flow of 0.3 ml/min and the injection volume was 2 µl. Gradient conditions for the analysis of the active substances of both PPPs can be found in the supplementary information (Supplementary Tables 1, 2).

Mass analysis in positive ESI mode was carried out using an AB Sciex 6500 QTRAP system (Applied Biosystems, Toronto, Canada) operated in the scheduled multiple-reaction-monitoring mode. The following parameters were used: nebulizing gas/ion source gas 1 (GS1) 20 psi and drying gas/ion source gas 2 (GS2) 50 psi, ionspray voltage (IS) 5500 V, entrance potential (EP) 10 V, collision gas (CAD) medium, curtain gas (CUR) 40 psi, and source temperature (TEM) 400 °C. Isolation and fragmentation of the parent ions were done, for each active substance (azoxystrobin, cyproconazole, epoxiconazole and fluxapyroxad). Optimization of declustering potential (DP), collision energy (CE) and collision cell exit potential (CXP) was done for each m/z transition, in order to maximize obtainable intensities. Target scan time was set to 2.3251 s and scheduled MRM detection window was set to 60 s. Detection parameters are displayed in Table 2(Tab. 2). Using the ion ratio as confirmatory parameter, two m/z transitions with the highest intensity were obtained for each analyte. Each sample was injected twice. Analyst software was used for data acquisition and processing, and Multiquant Software was used for data analysis. 

### Statistical analysis and modeling

Data were graphically visualized using the Graphpad Prism software (version 9.3.1).

Statistical analyses were performed with SigmaPlot (version 14) and the R software (version). Error bars represent the standard deviation of the mean. 

For cell viability, concentration data were transformed using X=log(X) and then normalized to the solvent control. Error bars indicate standard deviation, n=2 biological replicates each performed with six technical replicates. For gene expression analysis p-values were adjusted by the FDR-method (Benjamini and Hochberg, 1995[4]).

## Results

### Cytotoxicity 

**Figure 1:**
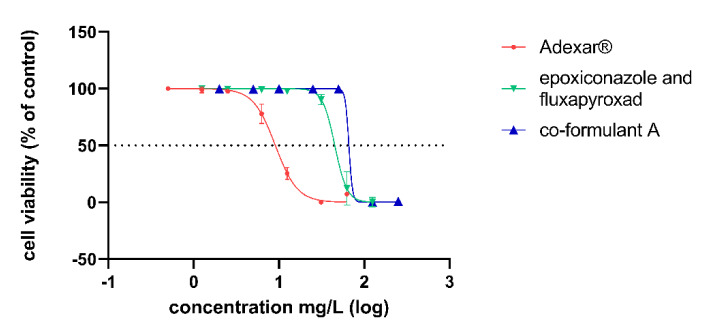
Cytotoxic effects of Adexar^®^ (red); its active substances mix (epoxiconazole and fluxapyroxad (green)); and a co-formulant of Adexar® (blue) on HepaRG cells after 24 h treatment obtained from NRU assay. Concentration data were transformed using X=log(X) and then normalized to the solvent control. Error bars indicate standard deviation, n=2 biological replicates each performed with 6 technical replicates. (Concentration of products expressed as the active substances' concentration within the product). Adexar® (a) contains the active substances fluxapyroxad and epoxiconazole in equimolar proportions (each 62.5 g/L).

**Figure 2:**
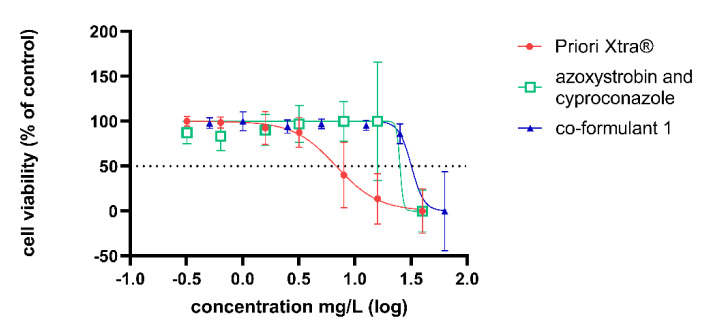
Cytotoxic effects of Priori Xtra^®^ (red); its active substances mix (cyproconazole and azoxystrobin, green) and a co-formulant (blue) of Priori Xtra^®^ on HepaRG cells after 24 h treatment obtained from NRU assay. Concentration data were transformed using X=log(X) and then normalized to the solvent control. Error bars indicate standard deviation, n=2 biological replicates each performed with 6 technical replicates. (Concentration of Priori Xtra^®^ expressed as the active substances' concentration within the product). Priori Xtra^®^ (b) contains ready-to-use 80 g/L cyproconazole and 200 g/L azoxystrobin.

Cell viability results showed a dose-response relationship with increasing concentrations of the substances in the case of Adexar^®^, Priori Xtra^®^ and their active substances mix in both the NRU and WST-1 assays on HepaRG cells after 24 h. Figure 1(Fig. 1) and 2(Fig. 2) summarize the results from the NRU assays. The WST-1 results are provided in the supplementary data. EC_50_ values obtained from the best fit values of the dose response curves generated from the results of the NRU assay are summarized in Table 3(Tab. 3). 

All available co-formulants of Adexar® were tested, but only one co-formulant which will be referred to as 'co-formulant A' not listed in MSDS, resulted in a dose-response curve with increasing concentration. The other co-formulants of Adexar®, 2-ethylhexyl-S-lactate and two other co-formulants not listed in the MSDS did not show observable cytotoxicity at the concentrations tested. Ethoxylated alcohol listed as a co-formulant in the formulation of Priori Xtra® also resulted in a dose-response relationship with increasing concentration on HepaRG cells. This co-formulant will be referred to as 'co-formulant 1'. Two other co-formulants of Priori Xtra^®^ did not decrease cell viability at the concentrations used. 

### Gene expression analysis

**Figure 3:**
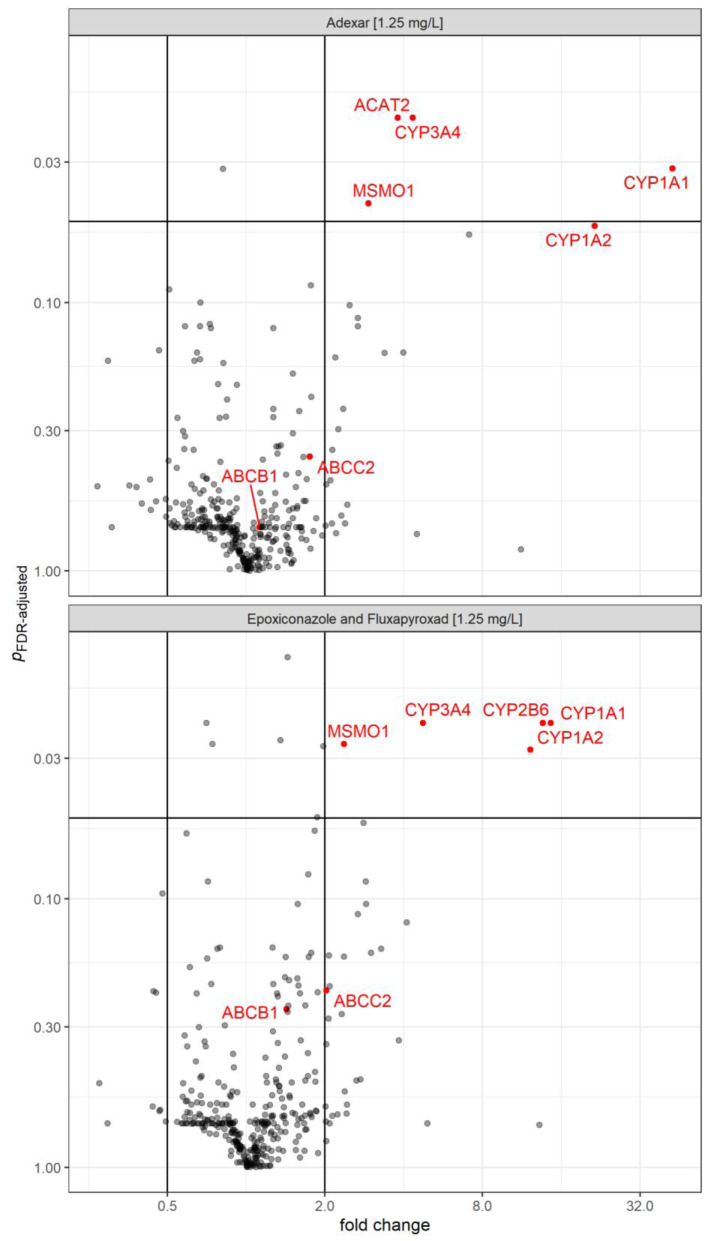
Gene expression changes (fold change) in HepaRG cells after 24 h treatment with Adexar® (1.25 mg/L (expressed as the active substances´ concentration within the product) in comparison to the solvent control and with the epoxiconazole and fluxapyroxad mix (1.25 mg/L each). Up and downregulation was considered relevant at <0.5 and >2-fold change in gene expression with significant gene transcripts indicated in red and labeled with gene symbols. P-values are adjusted by FDR-method (Benjamini and Hochberg, 1995). n=3 technical replicates

**Figure 4:**
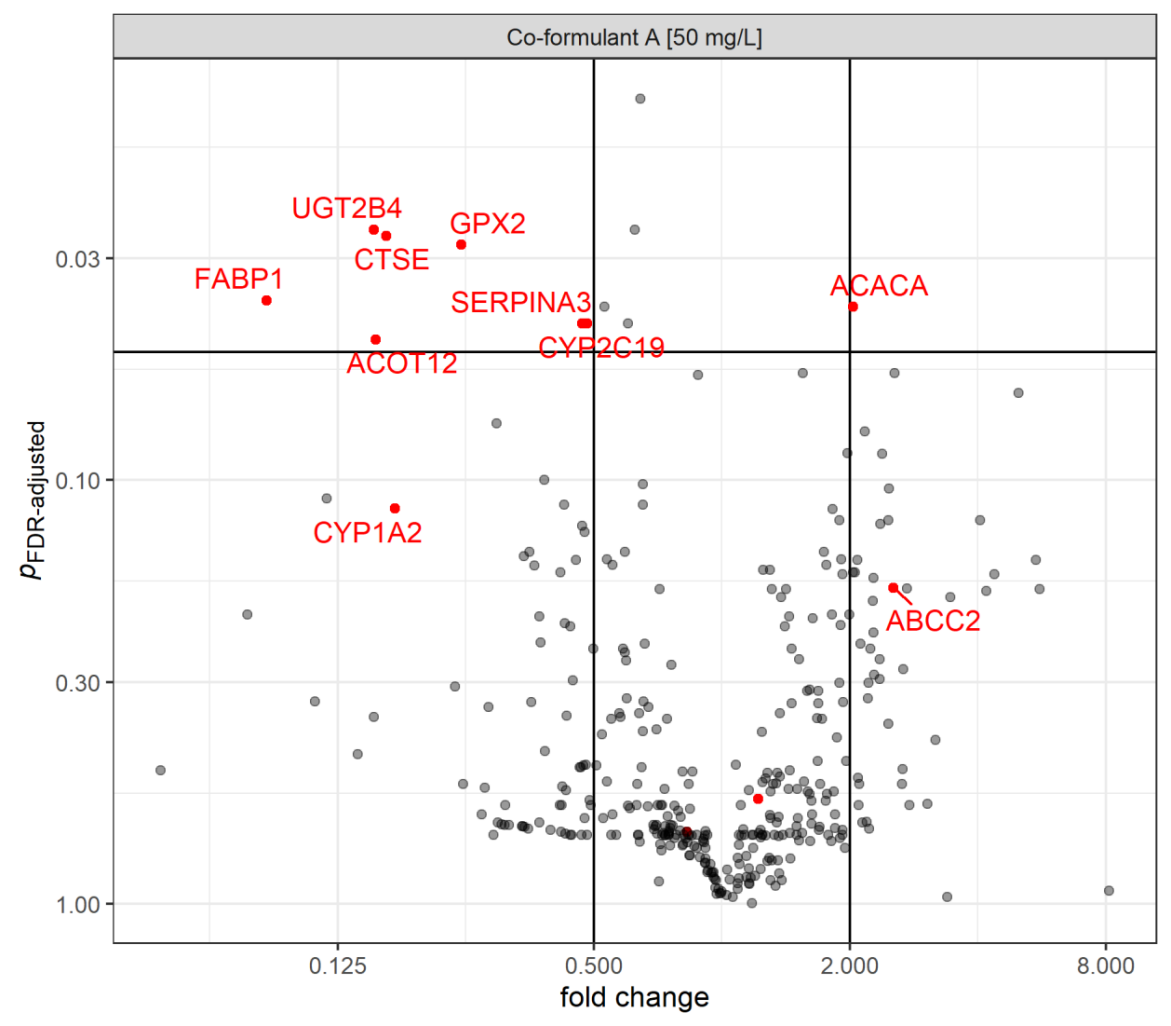
Gene expression changes (fold change) in HepaRG cells as a result of the 24 h treatment with co-formulant A of Adexar^®^ (50 mg/L) in comparison to the solvent control. Up and downregulation was considered relevant at <0.5 and >2-fold change in gene expression with significant gene transcripts indicated in red and labeled with gene symbols. P-values are adjusted by FDR-method (Benjamini and Hochberg, 1995). n=3 technical replicates

Gene expression analysis shows a similar gene expression profile between Adexar^®^ and its active substances' mix. For cells treated with Adexar^®^ significant changes after p-value correction observed were 4 out of 374 transcripts and the magnitudes of the differences were up to 32 fold regulation. *CYP1A1* and *CYP3A4* were upregulated, with the *CYP1A1* showing the largest upregulation of over 32-fold. Cells treated with Adexar^®^ and its active substances' mix similarly upregulated *CYP1A1*, *CYP3A4*, and *MSMO1* significantly, whereas *CYP1A2* and *CYP2B6* expression were significantly affected only by the active substances' mix. In addition, the expression of *ACAT2* was upregulated by Adexar^®^ and not by the mix. Figures 3(Fig. 3) and 4(Fig. 4) summarize the results. 

The gene expression of co-formulant A was investigated at the highest sub-cytotoxic concentration investigated (Figure 4(Fig. 4)). The gene expression analysis showed significant downregulation of the following gene transcripts: *UGT2B4*, *GPX2*, *CTSE*, *FABP1*, *SERPINA3*, *CYP2C19* and *ACOT12*. *ACACA* was significantly upregulated.

There were no significant gene expression changes observed in the treatments with Priori Xtra^®^ or with its active substances' mix (cyproconazole and azoxystrobin) at the concentrations selected.

GO term enrichment analysis suggested that upregulated DEGs significantly enriched in steroid biosynthesic processes in the case of Adexar^®^ (1.25 mg/L). Whereas, the epoxiconazole and fluxapyroxad mix (1.25 mg/L each) at the same concentration showed an influence. 

### Quantitative mass spectrometry-based immunoassays for CYP enzymes and transporters

**Figure 5:**
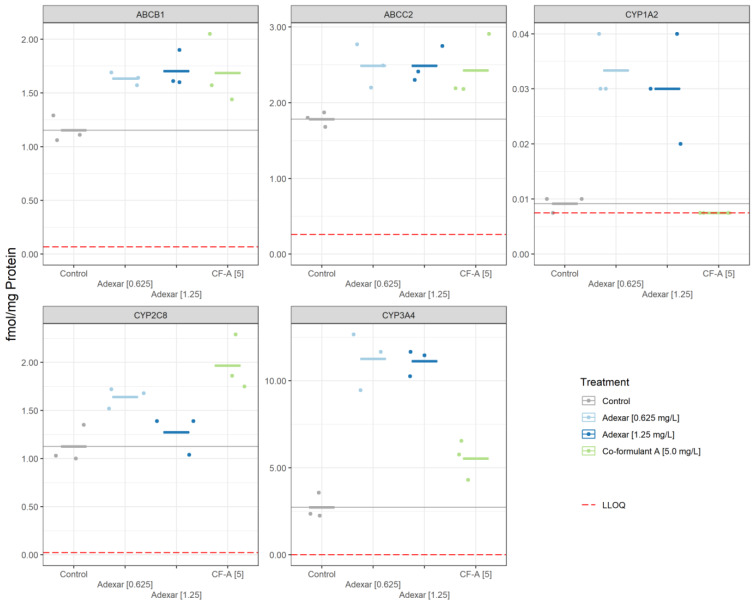
Treatment-related changes in protein concentration in HepaRG cells. Results were generated based on an MS-based immunoassay. Cytochrome P450 enzymes and the respective ABC transporter were quantified after 24 h treatment with the substances Adexar^®^ at 0.625 mg/L and 1.25 mg/L (expressed as the active substances´ concentration within the product); and co-formulant A of Adexar® (5.0 mg/L)

Protein quantification data reveals that the concentrations of several enzymes were influenced by the treatments. Figure 5(Fig. 5) summarizes the results. Specifically, CYP1A2, CYP3A4, and CYP2C8 proteins increased as a result of treatment with Adexar^®^ as compared to the solvent control (0.4 % DMSO). Co-formulant A induces the production of ABCB1 and ABCC2 transporters and CYP2C8 but not CYP1A2 and CYP3A4. 

### LC-MS/MS analysis

**Figure 6:**
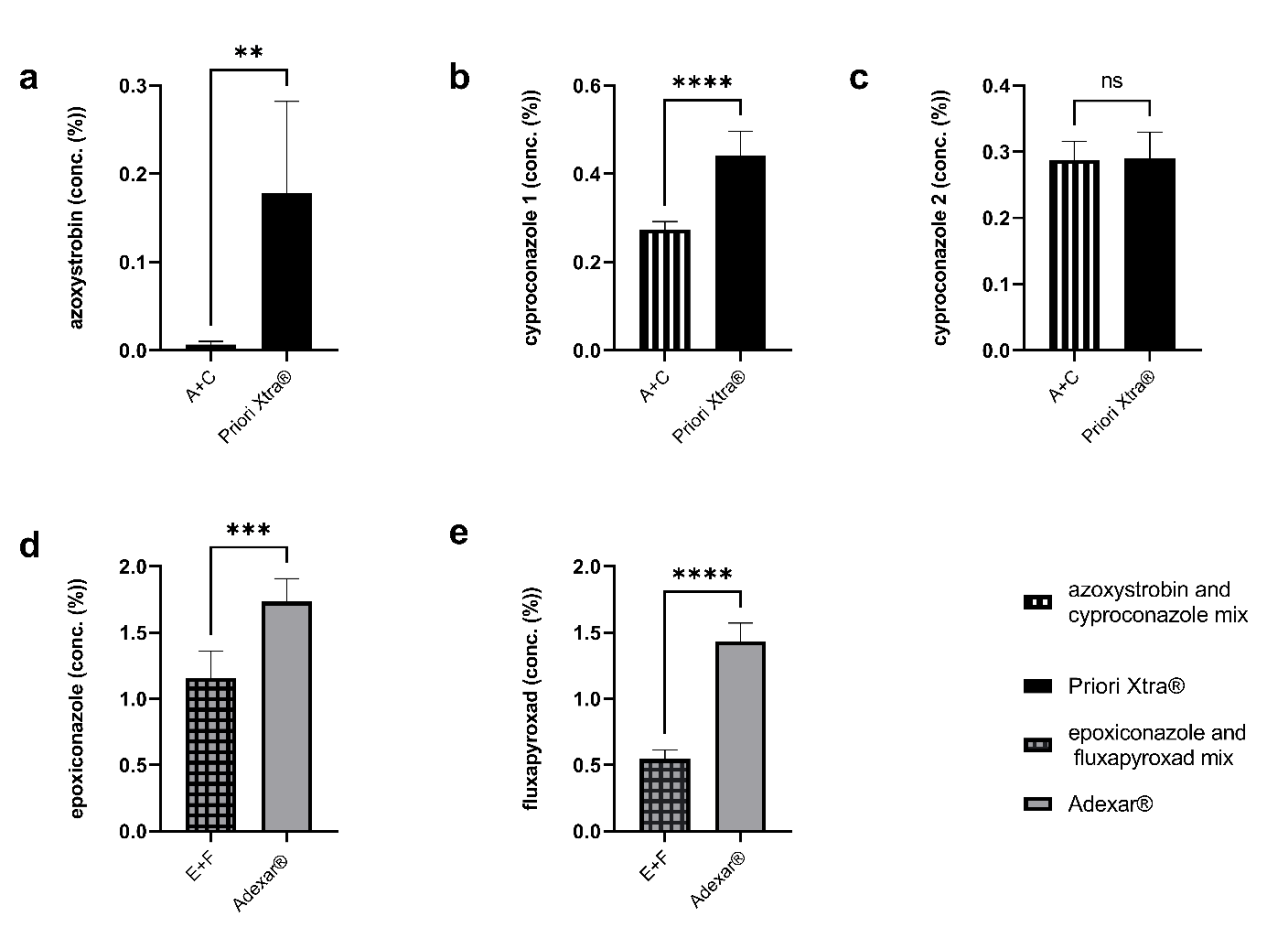
Active substances detected in HepaRG cells after 24 h treatment with Priori Xtra® and its active substances mix (a, b, c) and Adexar^®^ and its active substances mix (d, e) and using an LC-MS/MS system. n=3 biological replicates and each sample was injected twice. Significance shown with asterisks: P value ≤ 0.01 are summarized with two asterisks, P values less than 0.001 are summarized with three asterisks, and P values less than 0.0001 are summarized with four asterisks. Ns = not significant

The intracellular concentrations of the active substances were obtained in order to compare the concentrations of the active substances within the cells treated with the product, with the concentration of those treated with active substances' mix. Cells treated with Adexar^®^ and the active substance mix of Adexar^®^ were analyzed for epoxiconazole and fluxapyroxad. Similarly, cells treated with Priori Xtra^®^ and its active substances' mix were analyzed for cyproconazole and azoxystrobin. The percentage concentration of each active substance present in the medium, the cells, and PBS was calculated. The intracellular concentration values obtained are presented in Figure 6(Fig. 6). A higher percentage concentration of each active substance was detected in the HepaRG cells treated with the products as well as with the associated medium and PBS, compared to those treated with the mixtures of the respective active substances, except in the case of cyproconazole 2. The concentration of epoxiconazole detected in cells treated with Adexar® and cells treated with its respective active substances' mix (epoxiconazole and fluxapyroxad), was 1.77 % and 1.05 % respectively. A similar result was observed for fluxapyroxad which was detected at 1.43 % in the cells treated with Adexar^®^ and at 0.55 % with those treated with the active substances' mix. For the cells treated with Priori Xtra^®^ and its active substances mix azoxystrobin was detected at a 15 times higher concentration in the product (0.15 %) than in the mix (0.01 %). Cyproconazole is isomeric and was detected in two peaks as mentioned in the method. For the peak designated as cypro 1, values obtained were 0.46 % for the product and 0.28 % for the mix, whereas cypro 2 had similar values for the product and the mix at 0.30 % and 0.29 % respectively.

A higher percentage concentration of each active substance was detected in the HepaRG cells treated with the products as well as with the associated medium and PBS, compared to those treated with the mixtures of the respective active substances, except in the case of cyproconazole 2. The concentration of epoxiconazole detected in cells treated with Adexar® and cells treated with its respective active substances' mix (epoxiconazole and fluxapyroxad), was 1.77 % and 1.05 % respectively. A similar result was observed for fluxapyroxad which was detected at 1.43 % in the cells treated with Adexar^®^ and at 0.55 % with those treated with the active substances' mix. For the cells treated with Priori Xtra^®^ and its active substances mix azoxystrobin was detected at a 15 times higher concentration in the product (0.15 %) compared to in the mix (0.01 %). Cyproconazole is isomeric and was detected in two peaks as mentioned in the method. For the peak designated as cypro 1, values obtained were 0.46 % for the product and 0.28 % for the mix, whereas cypro 2 had similar values for the product and the mix at 0.30 % and 0.29 % respectively. 

## Discussion

The aim of this study was to address the observed differences between the toxicities of the products Adexar^®^ and Priori Xtra^® ^and their respective active substances mix on HepaRG cells, as well as, to determine the function of individual co-formulants in the outcomes. The previous study by Zahn et al. (2018[38]) with liver cells had revealed differences between the toxicities of the mentioned products and their respective active substances mix and this study serves as a follow-up.

In order to obtain dose-response curves, a narrow concentration range was selected for the cytotoxic evaluation of the substances. Concentrations of the products were expressed as the active substances' concentration within the product. This ensures that differences in the cytoxic effects on the cells could only be due to the co-formulants. Both PPPs showed increased cytoxicity as compared to their active substances as can be seen on Figure 1(Fig. 1) and 2(Fig. 2) as well as in Table 3(Tab. 3). This increased cytotoxicity of the PPPs confirms the findings in the previous study (Zahn et al., 2018[38]). We tested as many co-formulants available for each PPP. Four co-formulants of the product Adexar^®^ were tested and three co-formulants from Priori Xtra^®^. Figures 1(Fig. 1) and 2(Fig. 2) show that co-formulants exert toxic effects of their own, as one co-formulant of Adexar^®^ and one co-formulant of Priori Xtra^®^ investigated significantly decreased cell viability at concentrations comparable to that in the product. Concentrations of the co-formulants selected, were in the same ratio as they exist within the product formulation. 

Individual co-formulants have several functions, from use as a solvent to use as an emulsifier (Hazen, 2000[15]). Emulsifiers are surface active substances or surfactants that reduce interfacial tension between substances, allowing immiscible substances such as water and oil to form emulsions (Bart et al., 2013[3]). Co-formulant A of Adexar^®^ is an emulsifier but is not listed in the MSDS. Priori Xtra^® ^contains 20-30 % ethoxylated alcohol (here referred to as co-formulant 1) which is a non-ionic surfactant or emulsifier. Increased cytotoxicity of the products may be due to these surface active co-formulants. 

Through alteration of the toxicokinetic processes in the cells, the surface active co-formulants can cause an increase in the absorption or a decrease in the secretion of active substances. A recent study shows that the absorption of active substances was increased by surface active co-formulants (Karaca et al., 2021[20]). Similarly, our LC-MS/MS data show that after 24 h treatment, an increased concentration of each active substance was found in the cells treated with the PPPs as compared to the cells treated with active substances' mix for both Adexar^®^ and Priori Xtra^®^. Specifically, the percentage concentrations of epoxiconazole and fluxapyroxad of Adexar^®^ were significantly increased in the cells treated with Adexar^®^ compared to the cells treated with the active substances' mix. Similar results were obtained for azoxystrobin and one enantiomer of cyproconazole (denoted as cyproconazole 1). Although the indications are clear, our results provide only a snapshot of the event and do not demonstrate absorption or secretion. Additionally, we only investigated one concentration in our LC-MS/MS analysis. 

Transciptomic and proteomic data were used to gain a deeper understanding of the processes involved in the observed differences between the products and their active substances mix. We observed that the gene expression profile of cells treated with Adexar^®^ and those treated with its active substances' mix was similar. However, a few differences were noted. GO enrichment analysis suggests that cells treated with the epoxiconazole and fluxapyroxad mix (1.25 mg/L) influence steroid biosynthesis processes while cells treated with Adexar^®^ (1.25 mg/L) significantly enrich the steroid biosynthesis processes. The acyl coenzyme gene transcript 2 (*ACAT2*) has been identified as the major cholesterol esterifying enzyme in human livers. Inhibition of this enzyme has been suggested to have a positive impact in lowering plasma cholesterol levels in human patients (Parini et al., 2004[27]). *ACAT2 *was significantly upregulated in Adexar^®^ treated cells but not in those treated with the active substances' mix. In contrast to the combination of the active substances, in cells treated with Adexar^®^ *CYP2B6* was neither upregulated nor downregulated. There was no upregulation of *CYP2B6* in Adexar^®^ treated cells because *CYP2B6 *is induced by nuclear receptor CAR and transactivation of this nuclear receptor is reduced with Adexar^®^ treated cells (Zahn et al., 2018[38]; Gao and Xie, 2010[13]; Zhou, 2008[40]). These differential toxicodynamic changes in the HepaRG cells treated with Adexar^®^ in contrast to those treated with its active substances' mix can only be credited to the presence of co-formulants. Gene expression changes caused by Co-formulant A of Adexar^®^ were also investigated, albeit not at a concentration directly comparable to concentrations used for Adexar^® ^and its active substances' mix. The results show that co-formulants can affect the gene expression of the HepaRG cells. After p-value correction, no significantly affected gene transcripts were seen for either Priori Xtra^®^ or its active substances' mix.

As seen from the LC-MS/MS data, co-formulants role in the toxicity of the products may not only be due to toxicodynamic effects but also toxicokinetic. Here, proteomics results for Adexar^®^ demonstrate co-formulants' influence on the production of xenobiotic metabolizing enzymes and transporters. CYP2C8, an enzyme highly expressed in the liver and known to be involved in the metabolism of several drugs (Backman et al., 2016[2]), was induced by co-formulant A. CYP1A2 and CYP3A4 were not induced by co-formulant A.

ABC transporters such as ABCB1 and ABCC2 are ATP-dependent plasma membrane proteins implicated in the efflux of xenobiotics (Chedik et al., 2018[6]; Jedlitschky et al., 2006[19]). Hence, an induction of the transporters as observed from the proteomics results is expected to result in reduced intracellular accumulation of active substances. However, this was not the case, as shown from LC-MS/MS data. This can be explained by several simultaneous processes occurring in the cells as a response to the applied treatment, including both ATP- and non-ATP-dependent pathway processes. From our results, the sum of the processes resulted in an increase in the intracellular accumulation of the active substances in the cells treated with PPPs compared to the active substances' mix. The observed increased cytotoxicity of PPPs can be attributed to co-formulants' influence on the kinetic interaction of the active substances and the cells. 

In summary, co-formulants affect the toxicity of PPPs. As shown, Adexar^®^ and Priori Xtra^®^ are more cytoxic than their respective active substances' mix. The co-formulants result in a net increase in the intracellular concentrations of active substances in the PPPs treated cells as compared to those treated with active substances' mix. Gene expression profiles of HepaRG cells are altered in the presence of co-formulants, which also influence the production of xenobiotic metabolizing enzymes as well as ABC transporters. Therefore, co-formulants' contribution to the toxicity of PPPs should be more comprehensively investigated, taking into account potentiation of the PPPs' effect due to kinetic interaction. 

## Declaration

### Funding

This work was funded and supported by the German Federal Institute for Risk Assessment. 

### Conflict of interest

The authors declare to have no conflict of interest.

### Acknowledgments 

The authors would like to express gratitude to Dr. Corinna Kuerbis for allowing us to use the LC-MS/MS. We would also like to thank Annemarie Richter for technical support. 

## Supplementary Material

Supplementary information

Supplementary data

## Figures and Tables

**Table 1:**
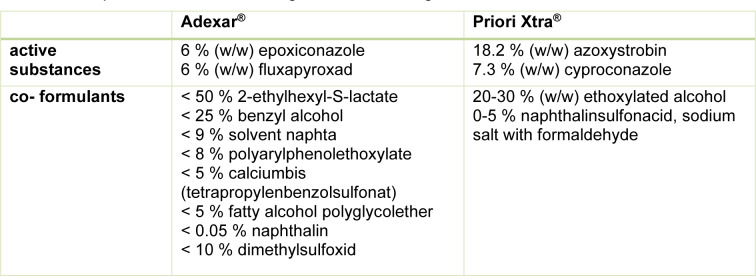
Composition information on ingredients according to MSDS of Priori Xtra^®^ and Adexar^®^

**Table 2:**
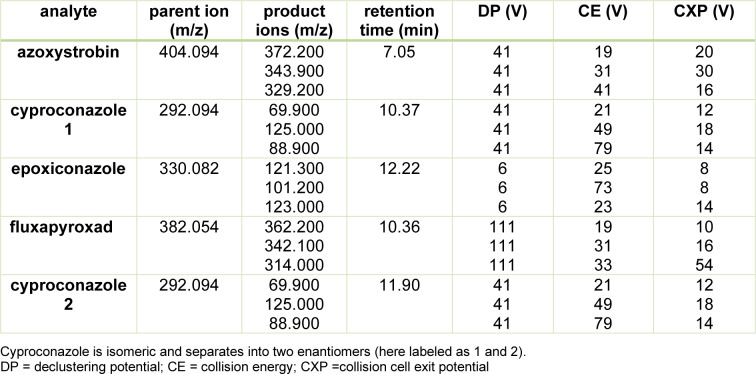
Parameters of detection

**Table 3:**
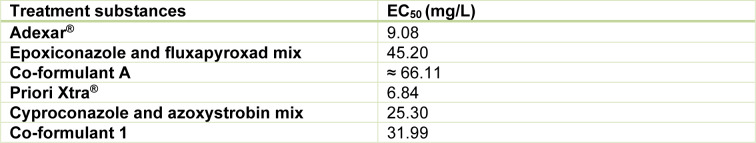
EC_50_ values obtained from best fit values of a non-linear dose-response curve of Adexar^®^, Priori Xtra^®^ and their active substances mix; co-formulant A; co-formulant 1 treatment on HepaRG cells after 24 h incubation
