# Supplementary material for: Mixture effects of co-formulants and two plant protection products in a liver cell line
Source: EXCLI J. 2023 Feb 13;22:221–36. doi: 10.17179/excli2022-5648 (PMC10043434; doi:10.17179/excli2022-5648)
Supplement: Supplementary information [file EXCLI-22-221-s-001.pdf]

EXCLI Journal 2023;22:221-236 – ISSN 1611-2156
Received: December 06, 2022, accepted: February 06, 2023, published: February 13, 2023

Supplementary information to:

Original article:

MIXTURE EFFECTS OF CO-FORMULANTS AND TWO PLANT PROTECTION PRODUCTS IN A LIVER CELL LINE

Katreece Feiertag, Mawien Karaca, Benjamin Fischer, Tanja Heise, Denise Bloch, Tobias Opialla, Tewes Tralau, Carsten Kneuer, Philip Marx-Stoelting*

German Federal Institute for Risk Assessment, Department Pesticides Safety, Berlin, Germany

* **Corresponding author:** Philip Marx-Stoelting, German Federal Institute for Risk Assessment, Department Pesticides Safety, Max-Dohrn-Str. 8-10, 10589 Berlin, Germany. Tel.: +49 30 1841226600, E-mail: Philip.Marx-Stoelting@bfr.bund.de

https://dx.doi.org/10.17179/excli2022-5648

This is an Open Access article distributed under the terms of the Creative Commons Attribution License (http://creativecommons.org/licenses/by/4.0/).

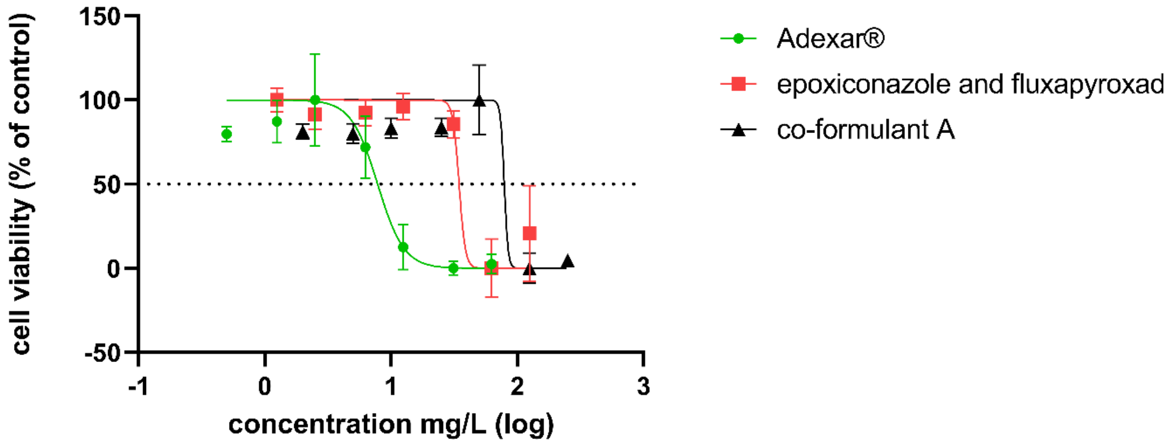

Supplementary Figure 1: Cytotoxic effects of Adexar® (green); its active substances' mix (epoxiconazole and fluxapyroxad (red)); and a co-formulant of Adexar® (black) on HepaRG cells after 24 h treatment obtained from WST-1 assay. Concentration data were transformed using X=log(X) and then normalized to the solvent control. Error bars indicate standard deviation, n=2 biological replicates each performed with 6 technical replicates. (Concentration of products expressed as the active substances' concentration within the product). Adexar® (a) contains the active substances fluxapyroxad and epoxiconazole in equimolar proportions (each 62.5 g/L).

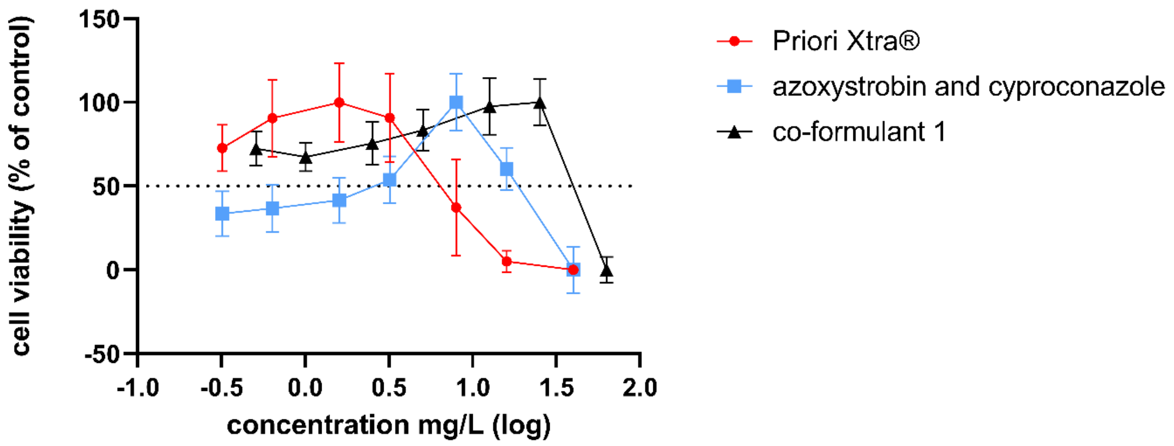

Supplementary Figure 2: Cytotoxic effects of Priori Xtra®(red); its active substances' mix (cyproconazole and azoxystrobin, (blue) and a co-formulant (black) of Priori Xtra® on HepaRG cells after 24 h treatment obtained from NRU assay. Concentration data were transformed using X=log(X) and then normalized to the solvent control. Error bars indicate standard deviation, n=2 biological replicates each performed with 6 technical replicates. (Concentration of Priori Xtra® expressed as the active substances' concentration within the product). Priori Xtra® (b) contains ready-to-use 80 g/L cyproconazole and 200 g/L azoxystrobin.
Only the concentration of cyproconazole is shown in case of both the mixture and the product.

EXCLI Journal 2023;22:221-236 – ISSN 1611-2156
Received: December 06, 2022, accepted: February 06, 2023, published: February 13, 2023

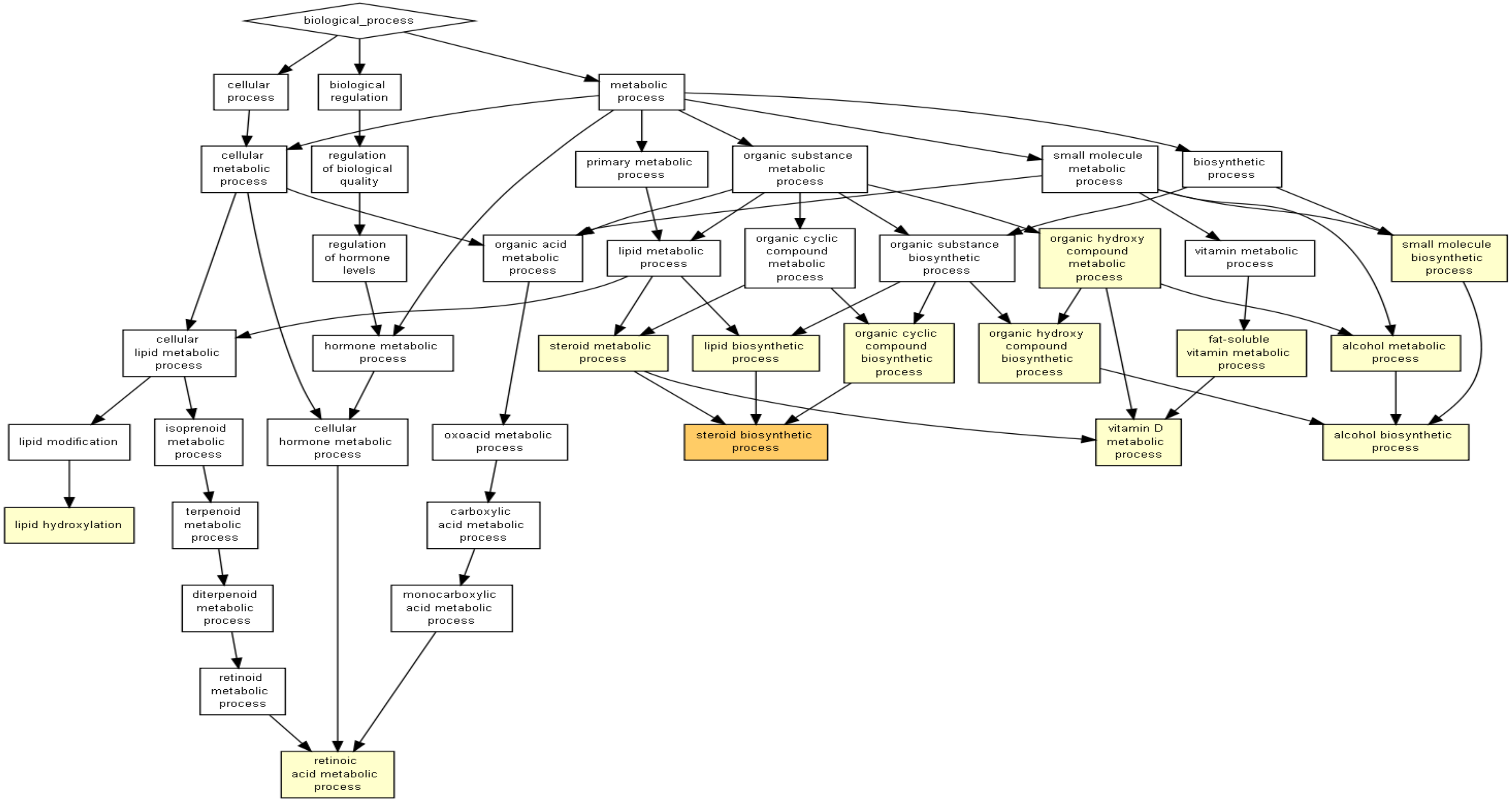

Supplementary Figure 3: GO Term result from gene expression data obtained from cells treated with Adexar® at 1.25mg/L

EXCLI Journal 2023;22:221-236 – ISSN 1611-2156
Received: December 06, 2022, accepted: February 06, 2023, published: February 13, 2023

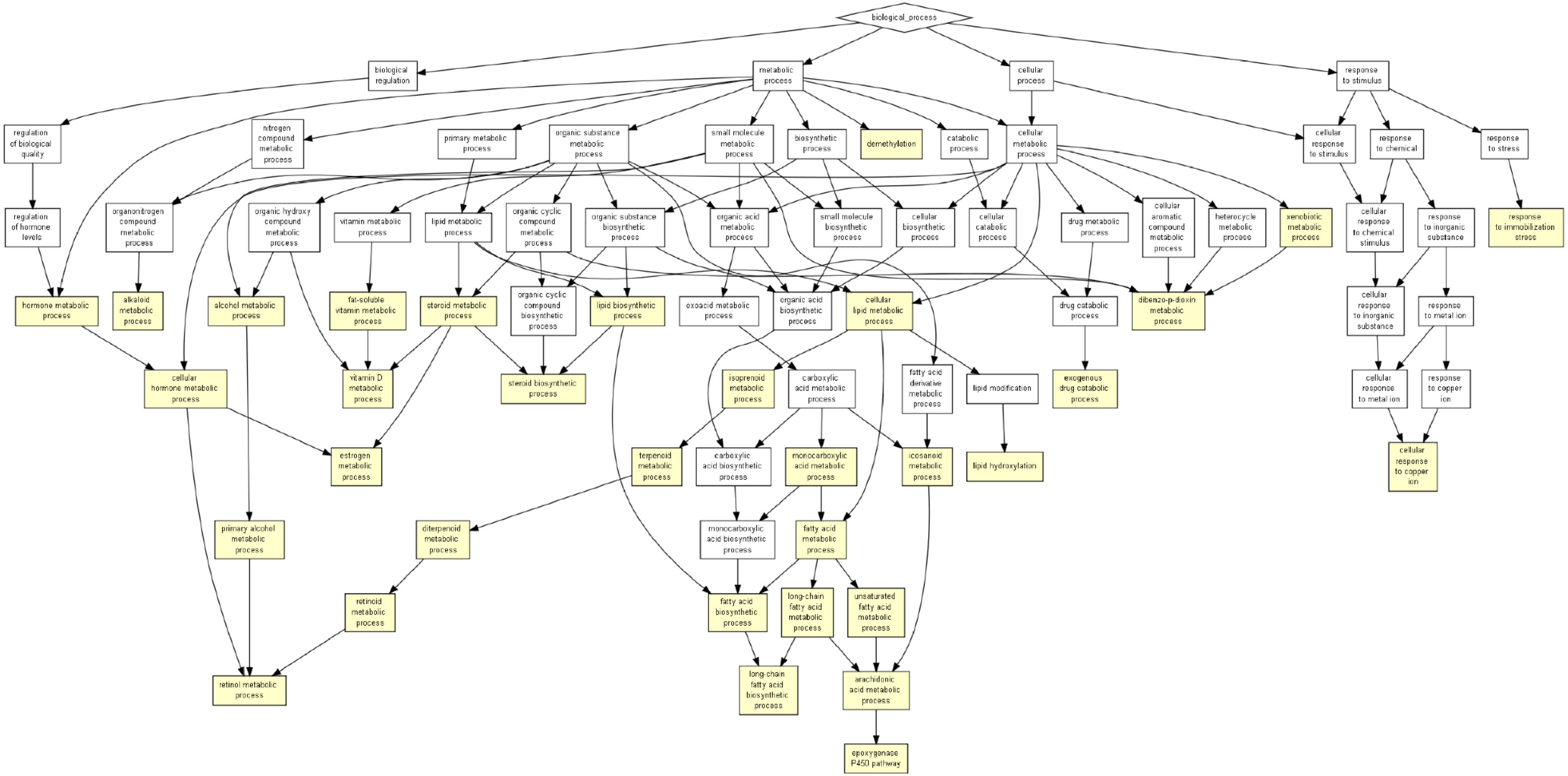

Supplementary Figure 4: GO Term result from gene expression data obtained from cells treated with Epoxiconazole and fluxapyroxad in combination at 1.25mg/L each.

EXCLI Journal 2023;22:221-236 – ISSN 1611-2156
Received: December 06, 2022, accepted: February 06, 2023, published: February 13, 2023

Supplementary Table 1: LC Pump Gradient conditions for the active substances azoxystrobin, cyproconazole, epoxiconazole and fluxapyroxad

Total time (min)	% A (0.2% (v/v) formic acid in water)	B % (Methanol)
0.00	40	60
14.00	30	70
14.05	40	60
20.00	40	60

Supplementary Table 2: Significantly affected gene transcripts after 24 h treatment with Adexar® (1.25 mg/L (expressed as the active substances' concentration within the product) and epoxiconazole and fluxapyroxad (1.25 mg/L each) in combination. Up and downregulation was considered relevant at <0.5 and >2-fold change in gene expression with significantly upregulated gene transcripts indicated in red.

Adexar®	epoxiconazole and fluxapyroxad
CYP3A4	CYP3A4
CYP1A1	CYP1A1
MSMO1	MSMO1
ACAT2	CYP2B6
	CYP1A2

Supplementary Table 3: Significantly affected gene transcripts after 24 h treatment with co-formulant A of Adexar® (50 mg/L) in comparison to the solvent control. Up and downregulation was considered relevant at <0.5 and >2-fold change in gene expression with significantly up and downregulated gene transcripts indicated in red and blue respectively.

Co-formulant A
ACACA
CYP2C19
ACOT12
SERPINA3
GPX2
CTSE
UGT2B4
FABP1
