# Supplementary material for: Mixture effects of co-formulants and two plant protection products in a liver cell line
Source: EXCLI J. 2023 Feb 13;22:221–36. doi: 10.17179/excli2022-5648 (PMC10043434; doi:10.17179/excli2022-5648)
Supplement: Supplementary data [file EXCLI-22-221-s-002.pdf]

EXCLI Journal 2023;22:221-236 – ISSN 1611-2156
Received: December 06, 2022, accepted: February 06, 2023, published: February 13, 2023

Supplementary data to:

Original article:

MIXTURE EFFECTS OF CO-FORMULANTS AND TWO PLANT PROTECTION PRODUCTS IN A LIVER CELL LINE

Katreece Feiertag, Mawien Karaca, Benjamin Fischer, Tanja Heise, Denise Bloch, Tobias Opialla, Tewes Tralau, Carsten Kneuer, Philip Marx-Stoelting*

German Federal Institute for Risk Assessment, Department Pesticides Safety, Berlin, Germany

* **Corresponding author:** Philip Marx-Stoelting, German Federal Institute for Risk Assessment, Department Pesticides Safety, Max-Dohrn-Str. 8-10, 10589 Berlin, Germany. Tel.: +49 30 1841226600, E-mail: Philip.Marx-Stoelting@bfr.bund.de

https://dx.doi.org/10.17179/excli2022-5648

This is an Open Access article distributed under the terms of the Creative Commons Attribution License (http://creativecommons.org/licenses/by/4.0/).

Received: December 06, 2022, accepted: February 06, 2023, published: February 13, 2023

Raw data tables concerning Figure 1: Cytotoxic effects of Adexar® (red); its active substances' mix (epoxiconazole and fluxapyroxad (green)); and a co-formulant of Adexar® (blue) on HepaRG cells after 24 h treatment obtained from NRU assay. Concentration data were transformed using X=log(X) and then normalized to the solvent control. Error bars indicate standard deviation, n=2 biological replicates each performed with 6 technical replicates. (Concentration of products expressed as the active substances' concentration within the product). Adexar® (a) contains the active substances fluxapyroxad and epoxiconazole in equimolar proportions (each 62.5 g/L).

Table 1: *Flourescent readings from Neutral red assay of HepaRG cells after 24 h treatment with Adexar® (1), epoxiconazole and fluxapyroxad in combination (2) and a co-formulant of Adexar®, co-formulant A (3) in concentration ranges from 0.5 to 62.5, 1.25 to 125 and 2 to 250 mg/L respectively.*

Adexar®											
Concentration mg/L	62,5	31,25	12,5	6,25	2,5	1,25	0,5	Positive control	Solvent Control	Media control	Blank
Sample 1	1180	650	2083	3960	5420	5587	5801	354	5563	5729	263
	1138	424	2568	4467	5395	5112	5559	489	5609	5474	209
	799	867	1898	3882	5240	5407	5406	295	5326	5367	199
	565	650	1772	4140	5347	5537	5690	505	5559	5644	353
	525	111	1432	4052	5520	5369	5459	115	5640	5667	85
	628	168	2061	4329	5347	5443	5482	289	5543	5563	105
Sample 2	548	193	830	4022	5713	5805	5762	145	5772	5582	110
	773	506	1972	5121	5585	5840	5818	219	5838	5743	203
	1146	777	1828	5297	5777	5779	5819	249	5810	5731	108
	1277	641	1485	4576	5737	5751	5727	303	5717	5871	247
	948	484	2560	5442	5914	5917	5794	273	5784	5776	89
	720	222	1015	5098	5698	5821	5731	300	5659	5645	805

EXCLI Journal 2023;22:221-236 – ISSN 1611-2156
Received: December 06, 2022, accepted: February 06, 2023, published: February 13, 2023

Epoxiconazole and fluxapyroxad											
Concentration mg/L											
	125	62.5	31.25	12.5	6.25	2.5	1.25	Positive control	Solvent control	Media Control	Blank
Sample 1	307	1335	5047	5413	5418	5433	5271	110	5514	5501	84
	956	1734	4189	5019	5463	5457	5408	258	5670	5644	257
	619	1804	5043	5283	5368	5404	5357	136	5561	5692	323
	642	1839	4876	5302	5487	5321	5370	145	5626	5578	191
	807	2042	5023	5321	5189	5468	5596	191	5705	5656	97
	877	1201	4727	5186	5310	5299	5337	159	5418	5294	77
Sample 2	321	748	5365	5740	5752	5786	5807	170	5670	5678	99
	386	587	5440	5636	5626	5715	5651	255	5723	5732	129
	363	479	5568	5664	5748	5737	5895	229	5928	5799	82
	574	783	5573	5753	5633	5826	5788	202	6004	5868	90
	302	785	5344	5768	5837	5894	5905	164	6094	6058	319
	385	460	5090	5473	5672	5662	5660	314	5818	5757	145

EXCLI Journal 2023;22:221-236 – ISSN 1611-2156
Received: December 06, 2022, accepted: February 06, 2023, published: February 13, 2023

Co-formulant A											
Concentration mg/L	250	125	50	25	10	5	2	Positive control	Solvent control	Media control	Blank
Sample 1	178	206	5756	5728	5691	5637	5702	120	5589	5714	92
	308	157	5855	5737	5715	5717	5735	299	5716	5757	111
	182	154	5864	5743	5752	5664	5666	135	5786	5794	129
	186	176	5796	5768	5730	5719	5608	408	5798	5858	110
	166	147	5808	5768	5833	5773	5806	174	5770	5766	115
	167	149	5723	5659	5710	5719	5731	187	5719	5673	104
Sample 2	519	429	5653	5687	5674	5714	5652	426	5747	5636	155
	659	488	5481	5559	5618	5666	5686	618	5764	5697	143
	755	785	5556	5588	5601	5441	5630	624	5699	5739	183
	658	690	5464	5628	5681	5511	5595	556	5540	5661	551
	574	546	5368	5489	5380	5461	5422	425	5378	5202	257
	313	261	5468	5448	5589	5643	5695	260	5670	5586	160

EXCLI Journal 2023;22:221-236 – ISSN 1611-2156
Received: December 06, 2022, accepted: February 06, 2023, published: February 13, 2023

Raw data tables concerning Figure 1: Cytotoxic effects of Priori Xtra®(red); its active substances' mix (cyproconazole and azoxystrobin, green) and a co-formulant (blue) of Priori Xtra® on HepaRG cells after 24 h treatment obtained from NRU assay. Concentration data were transformed using X=log(X) and then normalized to the solvent control. Error bars indicate standard deviation, n=2 biological replicates each performed with 6 technical replicates. (Concentration of Priori Xtra® expressed as the active substances' concentration within the product). Priori Xtra® (b) contains ready-to-use 80 g/L cyproconazole and 200 g/L azoxystrobin.

Only the concentration of cyproconazole is shown in case of both the mixture and the product.

Table 2: *Flourescent readings from Neutral red assay of HepaRG cells after 24 h treatment with Priori Xtra® (1), cyproconazole and azoxystrobin in combination (2) and a co-formulant of Priori Xtra®, co-formulant 1 (3) in concentration ranges from 0.32 to 40, 0.32 to 40 and 2 to 250 mg/L respectively.*

Priori Xtra®											
Concentration mg/L											
	40	16	8	3.2	1.6	0.64	0.32	Positive control	Solvent control	Media control	Blank
Sample 1	223	2017	4506	5995	6159	6128	6128	247	6045	5992	148
	172	1846	4260	5971	6055	6139	6149	230	6145	6086	177
	217	2214	4538	5807	5992	5955	5733	215	5800	5916	126
	536	2667	4405	5924	6068	6093	5980	343	6101	6123	134
	227	1552	4612	5969	6107	6041	5972	206	6092	6055	152
	148	2047	4045	5823	5878	5789	5827	294	5834	5807	209
Sample 2	3384	2608	2255	4728	5866	5325	5411	2250	5499	5328	1557
	915	1040	2738	5749	5890	5829	5802	2604	5478	5445	3089
	1261	2619	2086	4206	3795	5113	5529	4713	5538	5833	240
	2819	1357	4013	3722	4276	5635	5793	3480	5239	5581	1843
	3453	3622	1883	5210	5500	5038	5271	2367	5288	5224	2729
	748	454	1093	4742	4620	5630	5536	1097	5220	5449	1435

EXCLI Journal 2023;22:221-236 – ISSN 1611-2156
Received: December 06, 2022, accepted: February 06, 2023, published: February 13, 2023

Azoxystrobin and cyproconazole											
Concentration mg/L	40	16	8	3.2	1.6	0.64	0.32	Positive control	Solvent control	Media control	Blank
Sample 1	4632	5561	5317	5272	5490	5606	5235	935	5718	5486	1246
	4588	5373	5365	5080	5308	5077	5252	2008	5367	5700	199
	4256	5828	5741	5382	5104	5275	5244	1405	5295	5018	2042
	4075	6807	5077	5530	5228	5454	5033	3217	5122	5360	1115
	4220	5122	5458	5603	5096	4960	5077	290	5028	5206	628
	4231	4433	4999	4934	4915	4965	5057	847	5072	5019	1408
Sample 2	4783	5718	5934	5892	5908	5876	5810	200	5796	5823	140
	4834	5573	5933	5877	5831	5822	5891	226	5815	5892	116
	4854	5653	5836	5848	5800	5840	5854	162	5757	5760	834
	4955	5690	5942	5867	5937	5658	5813	254	5930	5799	145
	5028	5635	5947	5853	5843	5786	5906	161	5941	5892	220
	4536	5345	5591	5651	5612	5628	5556	546	5653	5701	154

EXCLI Journal 2023;22:221-236 – ISSN 1611-2156
Received: December 06, 2022, accepted: February 06, 2023, published: February 13, 2023

Co-formulant 1											
Concentration mg/L	63.25	25.30	12.65	5.06	2.53	1.01	0.51	Positive control	Solvent control	Media control	Blank
Sample 1	1186	4716	5057	5202	5188	5522	5052	2067	1059	5307	2336
	4503	4900	5471	5038	5244	5039	5306	1501	5128	5618	2111
	5215	5161	5510	5046	5446	5301	5383	1470	5281	5116	1611
	3314	4745	5143	5497	4694	5258	5242	3407	5317	5386	762
	2353	5319	5263	5342	5147	5408	5679	1861	5341	5315	890
	1401	4321	5107	5429	5207	6150	5476	3862	5544	4708	1270
Sample 2	1611	5395	5651	5801	5785	5800	5654	177	5812	5611	191
	2026	5396	5631	5686	5618	5573	5612	116	5514	5639	81
	2061	5475	5520	5565	5683	5651	5494	106	5587	5500	252
	1709	5444	5631	5699	5585	5604	5545	146	5706	5682	96
	1225	5303	5247	5628	5340	5490	5622	123	5607	5642	107
	1208	5475	5586	5609	5630	5591	5631	114	5606	5635	71

EXCLI Journal 2023;22:221-236 – ISSN 1611-2156
Received: December 06, 2022, accepted: February 06, 2023, published: February 13, 2023

Raw data tables concerning Figure 5: Treatment-related changes in protein concentration in HepaRG cells. Results were generated based on an MS-based immunoassay. Cytochrome P450 enzymes and the respective ABC transporter were quantified after 24 h treatment with the substances Adexar® at 0.625 mg/L and 1.25 mg/L (expressed as the active substances' concentration within the product); and co-formulant A of Adexar® (5.0 mg/L).
Data shown are the mean +SD (3 technical replicates) for all enzymes quantified. The solvent control (0.4 % DMSO) is indicated as control.

Table 3: *Protein concentration of cytochrome P450 enzymes and ABC transporters generated using an MS-based immunoassay in cells treated with Adexar® at 0.625 mg/L and 1.25 mg/L (expressed as the active substances' concentration within the product); and co-formulant A of Adexar® (5.0 mg/L). LLOQ= lower limit of quantification; ULOQ= upper limit of quantification; LOQ= limit of quantification. Replicates indicated as a, b and c.*

[fmol/µg]	LLOQ [fmol/µg]	ULOQ [fmol/µg]	Control-a	Control-b	Control-c	Adexar-0.625-a	Adexar-0.625-b	Adexar-0.625-c	Adexar-1.25- a	Adexar-1.25-b	Adexar-1.25-c	co-for-mulant A a	co-for-mulant A b	co-for-mulant A c
Total amount of protein	NA	NA	0.58	0.575	0.675	0.6	0.583	0.678	0.689	0.687	0.527	0.561	0.518	0.603
CYP1A1	0.0075	50	< LOQ	< LOQ	< LOQ	0.01	0.01	0.01	0.02	0.02	0.02	< LOQ	< LOQ	< LOQ
CYP1A2	0.0075	50	0.01	0.01	< LOQ	0.03	0.04	0.03	0.04	0.03	0.02	< LOQ	< LOQ	< LOQ
CYP2B6	0.206	50	< LOQ	< LOQ	< LOQ	0.53	0.61	0.52	0.45	0.45	0.43	0.82	0.62	0.58
CYP2C8	0.023	50	1	1.35	1.03	1.72	1.68	1.52	1.39	1.39	1.04	2.29	1.86	1.75
CYP2C9	0.206	50	5.7	6.45	4.41	5.1	5.5	5.05	4.3	3.85	4.11	6.72	5.8	4.75
CYP2C18	0.0685	50	0.08	0.09	0.08	< LOQ	< LOQ	< LOQ	< LOQ	< LOQ	< LOQ	< LOQ	< LOQ	< LOQ
CYP2C19	0.023	50	0.83	0.56	0.62	0.42	0.38	0.42	0.3	0.4	0.46	1.24	0.75	0.9
CYP2D6	0.206	50	< LOQ	< LOQ	< LOQ	< LOQ	< LOQ	< LOQ	< LOQ	< LOQ	< LOQ	< LOQ	< LOQ	< LOQ
CYP2E1	0.023	50	0.64	0.95	0.57	0.75	0.89	0.81	0.63	0.67	0.48	0.99	0.86	0.65
CYP2F1	0.206	50	< LOQ	< LOQ	< LOQ	< LOQ	< LOQ	< LOQ	< LOQ	< LOQ	< LOQ	< LOQ	< LOQ	< LOQ
CYP3A4	0.0025	50	2.35	3.57	2.25	11.65	12.65	9.45	11.65	11.45	10.25	6.54	5.75	4.3
CYP3A5 LPNK	0.206	50	< LOQ	< LOQ	< LOQ	< LOQ	< LOQ	< LOQ	< LOQ	< LOQ	< LOQ	<LOQ	<LOQ	< LOQ
CYP3A7	0.206	50	<LOQ	< LOQ	< LOQ	< LOQ	< LOQ	< LOQ	< LOQ	< LOQ	< LOQ	< LOQ	< LOQ	< LOQ
CYP3A43	0.206	50	< LOQ	< LOQ	< LOQ	< LOQ	< LOQ	< LOQ	< LOQ	< LOQ	< LOQ	< LOQ	< LOQ	< LOQ
ABCB1	0.0685	50	1.11	1.29	1.06	1.57	1.64	1.69	1.9	1.61	1.6	2.05	1.57	1.44
ABCC2	0.2600	21	1.68	1.87	1.8	2.2	2.77	2.49	2.3	2.41	2.75	2.91	2.19	2.18
ABCC3	0.2775	67	1.12	1.02	0.79	0.93	1.06	1.29	0.9	1.09	0.89	1.29	0.96	1.13

EXCLI Journal 2023;22:221-236 – ISSN 1611-2156
Received: December 06, 2022, accepted: February 06, 2023, published: February 13, 2023

Raw data tables concerning Figure 6: Active substances detected in HepaRG cells after 24 h treatment with Adexar® and its active substances' mix (epoxiconazole and fluxapyroxad), and Priori Xtra® and its active substances' mix (azoxystrobin and cyproconazole) using an LC-MS/MS system. n=3 biological replicates and each sample was injected twice.

Table 4: *Concentration (ng/ml) of active substances detected in cells, medium and pbs (used for washing cells) after 24 h treatment with Adexar®, its active substances' mix (epoxiconazole and fluxapyroxad), and Priori Xtra® and its active substances' mix (azoxystrobin and cyproconazole). Epoxy= epoxiconazole, fluxa= fluxapyroxad, azoxy= azoxystrobin, cypro 1= cproconazole 1 and cypro 2= cyproconazole 2.*

		cells			cells injection 2			medium			medium injection 2			pbs			pbs injection 2		
mix	epoxi	721	592	614	718	589	598	728	679	695	727	672	691	127	202	214	132	210	227
	fluxa	341	281	295	298	245	249	427	341	338	387	300	298	79,1	113	116	67,8	97,2	102
product (Adexar®)	epoxi	884	1030	1108	873	1004	1087	1552	1622	1696	1551	1605	1693	666	657	561	698	666	590
	fluxa	718	784	855	647	711	772	1205	1310	1297	1126	1198	1204	578	599	548	524	528	508
mix	Azoxy	8,38	10,1	21,5	6,22	7,56	16,8	8,26	7,93	12,9	6,48	6,4	10,4	2,34	1,64	6,3	1,64	1,12	4,65
	cypro 1	216	212	214	192	185	196	890	908	908	813	822	831	259	239	298	234	215	271
	cypro2	219	213	238	188	183	204	601	613	634	525	536	561				158	146	196
product (Priori® Xtra)	Azoxy	107	102	219	0	79,1	178	413	469	446	361	414	390	107	131	116	84,6	106	91,5
	cypro 1	339	299	376	0	271	344	1769	1786	1769	1630	1660	1631	467	509	466	420	451	416
	cypro2	547	484	617	0	425	542	1729	1756	1730	1536	1561	1527	486	527	498	406	451	421

Zeros in red are missing data due to machine down time during the run.
